# Anti-Vascular Endothelial Growth Factor Therapy in Breast Cancer

**DOI:** 10.3390/ijms151223024

**Published:** 2014-12-11

**Authors:** Tina Bøgelund Kristensen, Malin L. T. Knutsson, Markus Wehland, Britt Elmedal Laursen, Daniela Grimm, Elisabeth Warnke, Nils E. Magnusson

**Affiliations:** 1 Department of Biomedicine, Pharmacology, Aarhus University, Wilhelm Meyers Allé 4, Aarhus C 8000, Denmark; E-Mails: tinakristensen@studmed.au.dk (T.B.K.); malin.knutsson@studmed.au.dk (M.L.T.K.); 2 Clinic for Plastic, Aesthetic and Hand Surgery, Otto-von-Guericke-University Magdeburg, Leipziger Str. 44, Magdeburg D-39120, Germany; E-Mails: markus.wehland@med.ovgu.de (M.W.); elisabeth.warnke@med.ovgu.de (E.W.); 3 Department of Oncology, Aarhus University Hospital, Nørrebrogade 44, Aarhus C 8000, Denmark; E-Mail: britt@biomed.au.dk; 4 Medical Research Laboratory, Department of Clinical Medicine, Aarhus University, Nørrebrogade 44, Aarhus C 8000, Denmark; E-Mail: nm@clin.au.dk

**Keywords:** breast cancer, anti-angiogenic therapy, biomarkers, vascular endothelial growth factor

## Abstract

Neo-angiogenesis is a critical process for tumor growth and invasion and has become a promising target in cancer therapy. This manuscript reviews three currently relevant anti-angiogenic agents targeting the vascular endothelial growth factor system: bevacizumab, ramucirumab and sorafenib. The efficacy of anti-angiogenic drugs in adjuvant therapy or as neo-adjuvant treatment has been estimated in clinical trials of advanced breast cancer. To date, the overall observed clinical improvements are unconvincing, and further research is required to demonstrate the efficacy of anti-angiogenic drugs in breast cancer treatments. The outcomes of anti-angiogenic therapy have been highly variable in terms of tumor response. New methods are needed to identify patients who will benefit from this regimen. The development of biomarkers and molecular profiling are relevant research areas that may strengthen the ability to focus anti-angiogenic therapy towards suitable patients, thereby increase the cost-effectiveness, currently estimated to be inadequate.

## 1. Introduction

Breast cancer is the second most common cancer worldwide with an estimate of 1.7 million diagnosed cases in 2012 [1]. Since 2008, there has been a marked increase in breast cancer by more than 20% [1]. The survival of the patients suffering from breast cancer is strongly associated with prognostic factors, including tumor size, hormone-receptor-profile and existence of metastases. The primary treatment of breast cancer is surgical removal of the tumor, either as a lumpectomy or a mastectomy. Preoperative analyses of tumor size and possible metastases to the sentinel lymph node are decisive for the choice of surgery and the following treatment. Adjuvant therapies, such as chemotherapy, radiation and anti-hormone therapy, targeted treatment against human epidermal growth receptor 2 (HER2) and anti-angiogenic therapy can be applied postoperatively or, in the case of advanced disease stages, when surgery is no longer an option [2,3]. It is estimated that 50% of breast cancer patients are fully cured by surgical removal of the tumor, and another 25% are cured by surgical resection followed by postoperative systemic chemotherapy. At the moment, trials are also investigating the effect of neo-adjuvant treatment of breast cancer to minimize spread and improve the conditions for curative surgical tumor removal [4,5]. Sufficient oxygen and nutrition supply is essential for tumor growth. To support tumor growth, a rapid increase in the formation of blood vessels is required. Tumor angiogenesis is a multistep process requiring signaling between tumor cells and several cell types within the tumor microenvironment. This leads to overexpression of pro-angiogenic factors by the tumor, such as vascular endothelial growth factor (VEGF), referred to as the “angiogenic switch” [6].

High levels of circulating VEGF are a well-established indicator of poor prognosis [7]. The VEGF family consists of several signal protein variants and their receptors. Among them, the VEGF-A and VEGF receptor (VEGFR) subtype 2 interaction is the predominant interaction in angiogenesis [8]. One of the main regulators of the VEGF expression is oxygen tension. Hypoxic conditions observed in the interior of solid tumors activates hypoxia inducible factor (HIF), which initiates the transcription of various cytokines, including VEGF, which is a potent endothelial mitogen and pro-angiogenic factor [6]. Tumor vasculature shows abnormal features being twisted, heterogeneous, irregular lumen and exhibit atypical branching. The pericytes and basement membranes are abnormal, as well [8], and high turnover of vessels, reduced perfusion and increased leakage are observed [9]. Anti-angiogenic therapy inhibits tumor vessel growth by interfering with the intracellular signaling of VEGF and VEGFR [9,10,11].

## 2. Anti-Angiogenic Therapy

### 2.1. Angiogenesis

#### 2.1.1. Definition of Angiogenesis

Angiogenesis has been used as a term since 1935. It was first introduced to describe the formation of new blood vessels in the placenta, but later, the term included the formation of new vessels in wound healing and in tumor growth, as well. In 1971, Judah Folkman hypothesized that targeting angiogenesis might be useful in treating cancer [12].

#### 2.1.2. Spouting, Intussusception, Vascular Mimicry, Vascular Co-Option and Regulation of These Processes

There are several modes of vessel formation. In the embryo, the *de novo* generation of blood vessels originates from a mesoderm-derived hemangioblast, a common stage in the development of endothelial cells (EC) and blood cells, which differentiate into angioblasts that come together to form a vascular labyrinth in a process called vasculogenesis [11]. Angiogenesis, the development of new blood vessels, is important in cancer development, since tumor growth is dependent on the sufficient supply of oxygen and nutrients. The diffusion from capillaries is unable to go beyond 100–200 μm; therefore, in order to sustain cell function and to survive, the tumor must recruit new vessels [13]. ECs have oxygen sensors and hypoxia-inducible factors. This gives the ECs the ability to adjust their shape [8]. Endothelial sprouting is the main mechanism of tumor angiogenesis. This process, which involves ECs, pericytes, stroma cells and the extracellular matrix (ECM), depends on the upregulation of vascular endothelial growth factors [6]. There are several pathways that regulate endothelial cell migration. VEGF has been shown to regulate the release of matrix metalloproteinases (MMPs) and urokinase plasminogen activators, both damaging the basal membrane and the extracellular matrix. It allows EC migration and sprouting [9]. During the sprouting process, specialized ECs take the lead in the formation of the sprouting vessel. This cell is called the tip cell. The tip cell guides the vessel towards the tumor with its hypoxic, avascular region. It is followed by a so-called stalk cell, which divides to elongate the sprouting vessel. The tip cells express VEGFR-2 and Delta-like ligand 4, which binds to NOTCH receptors on the stalk cells. This interaction downregulates the expression of VEGFR-2 on the stalk cells, which allows the tip cells to continue leading the sprout, while the stalk cells proliferate and form the lumen of the vessel [8]. After stabilization of the vessel walls, the cells become non-proliferating cells, called phalanx cells. These cells are interconnected by the molecules, VE-cadherin and claudins, which strengthen the vessel wall and create a permeability barrier. The phalanx cells also regulate blood flow and perfusion of the vessels and recruit pericytes and smooth muscle cells by producing platelet-derived growth factor-b. These smooth muscle cells cover the naked vessels for further stabilization of the new vessels [11]. Pericytes produce factors that suppress endothelial proliferation and promote cell survival, such as VEGF and ANG-1 [8]. Tumor vessels can grow in several other ways than sprouting, among them intussusception and vasculogenic mimicry. Intussusception is a process in which the vessel wall is stretched into the lumen and then splits the vessel into two. Vasculogenic mimicry is a process in which the tumor cells alter their gene expression patterns to switch to a more undifferentiated phenotype that allows them to line vessels themselves. The vasculogenic mimicry seems to be independent of some pro-angiogenic factors, such as VEGF, and therefore, anti-angiogenic therapies do not always inhibit the process [11]. The formation of new vessels for tumor growth and metastasis plays a key role in cancer research and in the development of new drugs targeting angiogenesis. However, blood supply may be obtained by vessel co-option, the use of existing vessels, without triggering angiogenesis [14]. The mechanism was so far mostly reported in highly vascularized tissues, such as brain, lung and liver [15]. Donnem *et al*. [15] published that in primary and metastatic lung cancer and liver metastasis from different primary origins, as much as 10%–30% of the tumors use this alternative blood supply. This mechanism offers a potential explanation for angiogenic drug resistance observed in anti-angiogenic therapy, although the impact of vessel co-option requires further research.

### 2.2. Vascular Endothelial Growth Factor (VEGF) Family

**Figure 1:**
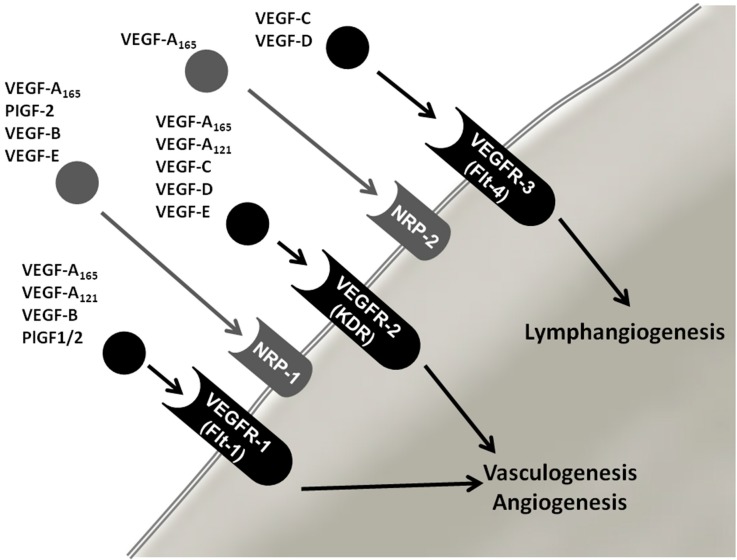
Overview of VEGF variants and their receptors. VEGFR, vascular endothelial growth factor (VEGF) receptor; PlGF-1/2, placental growth factor-1/2; NRP-1/2, Neuropilin-1/2.

VEGF is a very important factor involved in angiogenesis. The VEGF family includes eight members; VEGF-A (also referred to as VEGF), VEGF-B, VEGF-C, VEGF-E, VEGF-F, placental growth factor (PLGF)-1 and PLGF-2 [13]. The factors bind with different affinity and specificity to three tyrosine kinase receptors; vascular endothelial growth factor receptor VEGFR-1, VEGFR-2 (also known as FLK1) and VEGFR-3 (Figure 1). Vascular endothelial cells mainly express VEGFR-1 (binds VEGF-A, VEGF-B and PlGF) and VEGFR-2 (binds predominantly VEGF-A). VEGFR-3 is mainly found on lymphatic ECs and regulates the lymph-angiogenesis [10,16,17]. When binding to one receptor, the VEGFs stimulate dimerization of the receptor and initiate a signaling cascade that promotes ECs survival, growth and migration [18]. Furthermore, vessel permeability and endothelial progenitor cells are also stimulated. VEGF-A is the key factor in angiogenesis and primarily stimulates VEGFR-2 [13]. VEGF-A is also important in several physiological processes, including menstruation, ovulation, pregnancy, wound healing and maintenance of blood pressure [19,20,21,22]. It is a dimeric disulfide-bound glycoprotein that, due to splicing and posttranslational processing, can be composed of amino acid chains of various lengths. The most common isoform contains 165 amino acids and is overexpressed in several different human tumors [13,23]. In tumors, VEGF is produced by a variety of cells, including the tumor cells, the ECs and infiltrating myeloid cells. The interaction between the VEGF and its receptor will affect the EC in several different ways and will result in EC proliferation, migration, vascular permeability and invasion into the surrounding tissue and endothelial inflammation [9]. The main regulator of VEGF expression is oxygen tension. Under non-hypoxic conditions, the prolyl hydroxylase domain (PHD) protein hydroxylates HIF. When hydroxylated, HIF is recognized by the von Hippel-Lindau tumor suppressor (VHL) and is destroyed by proteases. In hypoxic conditions, like during tumor growth, the PHD is inactive, which hinders degradation, and HIF translocates to the nucleus and initiates transcription of various genes, among them VEGF, platelet-derived growth factor (PDGF) and MMP-1. In the sprouting process, the VEGF induces both NOTCH-mediated proliferation in the stalk cells and hypoxic migration cues to the tip cell [6].

## 3. Overview of Anti-Angiogenic Drugs Targeting the VEGF System

**Figure 2:**
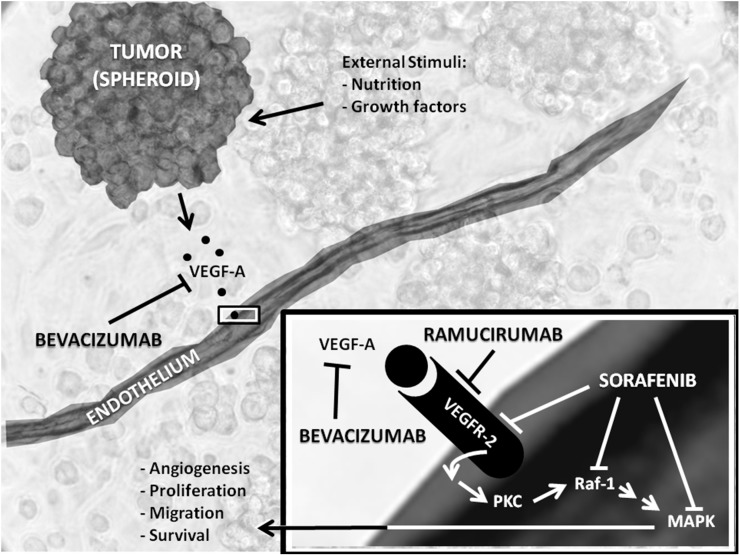
Overview of bevacizumab, ramucirumab and sorafenib and their inhibition of targets in anti-angiogenic treatment. VEGFR2, vascular endothelial growth factor (VEGF) receptor 2; Raf-1, proto-oncogene, serine/threonine kinase; PKC, protein kinase C; MAPK, mitogen activated protein kinase.

There are three major targets and groups of drugs available for anti-VEGF therapy: drugs interfering with the VEGF ligand, drugs interfering with the VEGFRs and drugs interfering with intracellular signaling of the VEGFRs [10,24]. Bevacizumab and ramucirumab are monoclonal humanized antibodies designed to inhibit the interaction between VEGF ligands and receptors [25,26], whereas sorafenib is a tyrosine kinase inhibitor (TKI) targeting VEGFRs, but also with an affinity for other tyrosine kinases, including platelet-derived growth factor receptor (PDGFR) [27]. An overview of the results from clinical trials of breast cancer using the anti-angiogenic drugs discussed in this review is given in Table 1. Furthermore, Figure 2 shows a schematic representation of the modes of action of these drugs.

**Table 1:** Overview of results of trials using bevacizumab, sorafenib and ramucirumab. For bevacizumab and sorafenib, progression-free survival (PFS), overall response rate (ORR), pathological complete response (pCR) and overall survival (OS) denote the effect of treatment with bevacizumab/sorafenib *vs.* without. NS, no significant improvement

Drug	Trial/Reference	Treatment	Drugs Used	PFS	ORR	pCR	OS
bevacizumab	AVF2119g	Second-line	Capecitabine +/− bevacizumab	NS	Significant	Not reported	NS
bevacizumab	ECOG-E2100	First-line	Paclitaxel +/− bevacizumab	Significant	Significant	Not reported	NS
bevacizumab	AVADO	First-line	Docetaxel +/− bevacizumab (arm treated with highest dosage bevacizumab)	Significant	Significant	Not reported	NS
bevacizumab	RIBBON-1	First-line	Capecitabine/taxane/Anthracycline based chemotherapy +/− bevacizumab	Significant	Significant	Not reported	NS
bevacizumab	RIBBON-2	Second-line	Capecitabine/taxane/gemcitabine/vinorelbine based chemotherapy +/− bevacizumab	Significant	Significant	Not reported	NS
bevacizumab	GBG44/clinicaltrials.gov ID: NCT00567554	Neoadjuvant therapy	Epirubicin/cyclophosphamide/docetaxel +/− bevacizumab	Not reported	Not reported	Significant	Data under way
bevacizumab	NSABP	Neoadjuvant therapy	Docetaxel/capecitabine/gemcitabine +/− bevacizumab	Not reported	Not reported	Significant	Data under way
bevacizumab	AVEREL	First-line	Trastuzumab/docetaxel +/− bevacizumab	NS	NS	Not reported	NS
sorafenib	SOLTI0701	First-line/second-line	Capecitabine +/− sorafenib	Significant	NS	-	NS
sorafenib	AB01B07	Second-line	Capecitabine and gemcitabine +/− sorafenib	Significant	NS	-	NS
sorafenib	NU07B1	First-line	Paclitaxel +/− sorafenib	NS	Significant	-	NS
sorafenib	FMB0701	First-line	Docetaxel and/or letrozole +/− sorafenib	NS	NS	-	NS
ramucirumab	TRIO-012	-	Doceraxel +/− ramucirumab	NS	NS	-	NS at interim analysis
ramucirumab	clinicaltrials.gov ID: NCT01427933	Second-line	Eribulin +/− ramucirumab	Not reported	Not reported	Not reported	Not reported

### 3.1. Bevacizumab

VEGF-A expression has been found to be upregulated in various human tumors, including breast cancer [27]. Bevacizumab is a monoclonal antibody that binds and inactivates soluble VEGF-A molecules, resulting in inhibition of VEGF-mediated angiogenesis [25]. Bevacizumab was mainly tested in combination with chemotherapeutic drugs and in the neoadjuvant therapy regimen [27,28]. These chemotherapeutics include capecitabine, paclitaxel, docetaxel, anthracyclines, gemcitabine and vinorelbine, which are approved chemotherapeutic drugs against breast cancer. The results of a variety of trials, including the randomized phase III trial of capecitabine compared with bevacizumab plus capecitabine in patients with previously-treated metastatic breast cancer [25] and the trial paclitaxel plus bevacizumab *vs.* paclitaxel alone for metastatic breast cancer (NCT00028990) [29], led to the approval of bevacizumab as an effective drug against breast cancer by the European Medicines Agency (EMEA) in March, 2007, and by the U.S. Food and Drug Administration (FDA) in December, 2008 [30]. In 2010 through 2014, trials, such as first-line trials “Avastin and Docetaxel” (AVADO), “Regimens in Bevacizumab for Breast Oncology” (RIBBON)-1 and “Maintenance capecitabine and bevacizumab *vs.* bevacizumab alone after initial first-line bevacizumab and docetaxel for patients with HER2-negative metastatic breast cancer” (IMELDA) [31,32,33] and second-line trials RIBBON-2 and “Bevacizumab plus chemotherapy *vs.* chemotherapy alone as second-line treatment for patients with HER2-negative locally recurrent or metastatic breast cancer after first-line treatment with bevacizumab plus chemotherapy” (TANIA) [34,35], confirmed the positive impact of bevacizumab on the endpoints of progression-free survival (PFS) and overall response rate (ORR), but also confirmed the missing impact on overall survival (OS). Furthermore, serious adverse effects were also demonstrated in these trials. As a consequence, the FDA withdrew their earlier approval of bevacizumab as an effective anti-angiogenic agent against HER2-negative metastatic breast cancer. In contrast, the EMEA maintained their approval [36]. The main results until now are that treatments with bevacizumab in combination with chemotherapy have significantly improved the endpoints of ORR, PFS and pathological complete response (pCR) *vs.* patients treated with chemotherapy plus placebo. According to the AVADO trial, bevacizumab improved efficacy, including one-year OS rates (71% *vs.* 65%). The PFS was 8.1 months with bevacizumab *vs.* 5.4 months with chemotherapy alone [31]. Several earlier trials showed a prolonged PFS (Table 1). Two trials did not show improved ORR and PFS; the AVF2119g trial demonstrated an insignificant effect on the endpoint of PFS after adding bevacizumab to capecitabine as a second line treatment of metastatic breast cancer. Furthermore, the hazards ratio for investigator-assessed PFS in the AVEREL trial of HER2-positive metastatic breast cancer patients was not significantly affected (0.82; 95% CI: 0.65 to 1.02; *p* = 0.0775) [37]. Median OS exceeded 38 months in both arms. There was no difference between the treatment arms in OS [37]. Positive results for PFS and OS were found in the IMELDA trial [33]. In the ongoing TANIA (clinicaltrials.gov ID: NCT01250379) trial [35], the magnitude of improvement in progression-free survival (stratified HR 0.75, 95% CI: 0.61–0.93; log-rank *p* = 0.0068) is very similar to that noted in the RIBBON-2 trial. Continuous second-line combination treatment with bevacizumab and chemotherapy significantly improved PFS compared to bevacizumab, for patients with metastatic or locally-recurrent breast cancer. In addition, in the IMELDA study, OS was significantly improved, 39.0 *vs.* 23.7 months [33]. Adverse effects of bevacizumab include hypertension, bleeding events, proteinuria and sensory neuropathy [38]. In many cases toxicity or progression of disease forced patients to discontinue therapy. The subpopulations of patients discontinuing therapy were higher for those patient groups treated with bevacizumab *vs.* placebo treatment patient groups [30]. Currently ongoing trials of bevacizumab in the therapy of breast cancer are listed in Table 2.

### 3.2. Ramucirumab

Ramucirumab is a humanized monoclonal antibody against the VEGF-binding domain of the VEGFR-2, which is found to be overexpressed in many human breast cancers and seems be an important receptor facilitating VEGF-mediated angiogenesis [38]. Ramucirumab binds to VEGF-2 with strong affinity and inhibits interaction between tumor-produced VEGF and VEGFR-2 [39]. A phase I study from 2010 examined the effect of ramucirumab against different malignant tumors and revealed promising data for ramucirumab in anti-angiogenic therapy. Keeping in mind that the study population was relatively small and heterogeneous, ramucirumab decreased tumor perfusion and vascularity in 69% of evaluable patients [39]. Adverse effects associated with ramucirumab are similar to those for bevacizumab [26]. At the moment, a phase III trial on ramucirumab in combination with chemotherapy is ongoing [40]. The trial started in 2009 and recruited patients with yet untreated HER2-negative metastatic breast cancer. The patients are randomized to receive docetaxel plus ramucirumab *vs.* docetaxel plus placebo in three-week-cycles throughout the follow-up period. The study will be published in December 2015, and will reveal the potential effects of ramucirumab in combination with docetaxel for first-line treatment of HER2-negative metastatic breast cancer. A phase II open-label study of 141 unresectable patients randomized to receive eribulin monotherapy or eribulin plus ramucirumab in three-week cycles was completed in September 2013. Results of both safety and efficacy await publication (clinicaltrials.gov ID: NCT01427933).

**Table 2:** Current protocols studying bevacizumab intervention in breast cancer. The table is based on studies registered at ClinicalTrial.gov. [41] and specific search criteria: phase I–IV, open studies, exclude unknown status, interventional studies. Condition: breast cancer. Intervention: bevacizumab, all ages, all gender, start date: 1 January 2013–1 January 2014.

Protocol Title	Phase	Condition	Intervention	Clinicaltrials.gov
Patient Preference for Everolimus in Combination with Exemestane or Capecitabine in Combination with Bevacizumab (IMPROVE)	IV	Advanced (Inoperable or Metastatic) HER2-negative Hormone Receptor Positive Breast Cancer	Everolimus + Exemestane *vs*. Capecitabine + Bevacizumab	NCT02248571
Trastuzumab or Bevacizumab with Combination Chemotherapy in Treating Patients with Stage II–III Breast Cancer	II	Stage II Breast Cancer; Stage IIIA Breast Cancer; Stage IIIB Breast Cancer; Stage IIIC Breast Cancer	Trastuzumab *vs.* Bevacizumab + Chemotherapy (Docetaxel; Carboplatin; Doxorubicin Hydrochloride; Cyclophosphamide, Paclitaxel)	NCT01959490
Bevacizumab in Combination with Chemotherapy in the Neo-adjuvant Setting for HER2 (−) Breast Cancer	II	Breast Cancer	Neoadjuvant Treatment of Bevacizumab + Chemotherapy (5-Fluorouracil, Epirubicin; Cyclophosphamide; Docetaxel)	NCT01985841
Bevacizumab, Etoposide and Cisplatin Followed by Whole Brain Radiotherapy in Breast Cancer with Brain Metastases	II	Breast Cancer; Brain Metastases	BEEP (bevacizumab preconditioning followed by etoposide and cisplatin) Regimen Prior to Radiotherapy	NCT02185352
Safety and Efficacy Study of Eribulin in Combination with Bevacizumab for Second-line Treatment HER2-MBC Patients (GIM11-BERGI)	II	Metastatic Breast Cancer; Human Epidermal Growth Factor 2 Negative Carcinoma of Breast	Bevacizumab and Eribulin	NCT02175446
Intrapleural Bevacizumab After Pleural Drainage in the Context of Breast Cancer	I	Pleural Effusion, Malignant; Breast Cancer	Bevacizumab	NCT02250118
Bevacizumab Plus Paclitaxel Optimization Study with Interventional Maintenance Endocrine Therapy in Advanced or Metastatic ER-positive Human Epidermal Growth Factor Receptor 2(HER2)-Negative Breast Cancer (BOOSTER)	II	Metastatic Breast Cancer	Weekly Paclitaxel + Bevacizumab *vs*. Weekly Paclitaxel + Bevacizumab Followed By Hormone Therapy + Bevacizumab Then back to Weekly Paclitaxel + Bevacizumab	NCT01989780
Triple-B Study; Carboplatin-cyclophosphamide *vs.* Paclitaxel with or without Bevacizumab as First-line Treatment in Advanced Triple Negative Breast Cancer	II	Breast Cancer	Carboplatin/Cyclophosphamide *vs.* Carboplatin/Cyclophosphamide + Bevacizumab *vs.* Paclitaxel *vs.* Paclitaxel + Bevacizumab	NCT01898117
Phase I Study of Lurbinectedin (PM01183) in Combination with Paclitaxel, with or without Bevacizumab, in Selected Advanced Solid Tumors	I	Breast Cancer; Ovarian Cancer; Gynecological Cancer; Head and Neck Carcinoma; Non-Small-Cell-Lung Cancer; Small-Cell-Lung Cancer; Non-Squamous-Cell-Lung Cancer	PM01183 + Paclitaxel *vs*. PM01183 + Paclitaxel + Bevacizumab	NCT01831089

### 3.3. Sorafenib

Sorafenib is a tyrosine kinase inhibitor and exerts an anti-proliferative and anti-angiogenic activity by blocking the intracellular signal transduction of VEGFR-2 in endothelial cells. Monotherapy using sorafenib has not shown significant results in breast cancer [42]. Some trials suggest that due to the fact that sorafenib targets angiogenesis at multiple steps, the agent may be able to affect the pathways involved in the case of bevacizumab-resistance. This theory was tested by applying sorafenib to second-line treatments of chemotherapy to patients who had failed therapy with bevacizumab. The results of the trial demonstrated a significant prolongation in PFS, when sorafenib was added to gemcitabine/capecitabine [43]. Four relevant phase II trials, known as the Trials to Investigate the Efficacy of Sorafenib (TIES) have tested the effect of sorafenib in patients with HER2-negative advanced or metastatic breast cancer [42]. The common conclusion of the trials is that sorafenib has anti-tumor activity and improves PFS and ORR, when it is applied in combination with certain chemotherapeutic drugs. None of the trials demonstrated a beneficial impact of sorafenib on OS [42]. Multiple adverse effects have been observed with protein kinase inhibitors and involve almost every organ of the body. Most frequent adverse effects are hypertension, anemia, rash, neutropenia, thrombocytopenia, fever, infections and fatigue. Further trials are needed to assess the beneficial anti-angiogenic effects of sorafenib. A phase III trial on capecitabine +/− sorafenib was initiated in 2010. The primary endpoint will be PFS, and secondary focuses will be the improvements of tolerability by dosage adjustments and the search for potential biomarkers [42].

### 3.4. Adverse Effects

The dosage of anti-cancer therapy is limited by its adverse effects and is usually not adjusted according to the lowest dose of the therapeutic window, as is the case in most other pharmacologic therapy [44]. Generally observed adverse effects, in relation to anti-angiogenic therapy, are hypertension, congestive heart failure, proteinuria caused by renal failure, bone marrow depression, rash and sensory neuropathy. A recent retrospective analysis has estimated a prevalence of 38% of bevacizumab-induced hypertension in breast cancer patients. Furthermore, the study suggested that development of hypertension during treatment might be a marker for the effect of bevacizumab [45].

## 4. Biomarkers

Biomarkers are able to define the subpopulation of breast cancer patients that benefits from anti-angiogenic therapy and makes it possible to target treatment therapies towards susceptible patients, which will increase the efficacy of anti-angiogenic therapy [46]. Especially, biomarkers related to treatment with bevacizumab have been intensively investigated. The most debated possible biomarkers involve HER2-receptor status, polymorphisms in genes, such as *BRCA1/2*, *TP53* and *PTEN* [47,48,49], the level of plasma-VEGF-A and, recently, hypertension. The outcomes of research on HER2-receptor status are inconclusive. First, an exploratory subgroup analysis of the RIBBON-2 trial demonstrated a higher improvement of PFS in triple-negative breast cancer (TNBC) patients suggesting that TNBC and HER2-negative patients have the strongest benefit of bevacizumab treatment [34]. Correlation between the prognosis of anti-angiogenic therapy and HER2/estrogen receptor status has yet to be shown. In a retrospective study on the data of Eastern Cooperative Oncology Group (ECOG)-E2100 [29], specific single-nucleotide polymorphisms (SNPs) in genes of VEGF were identified that could be used to identify the subgroups of patients being most receptive for anti-angiogenic therapy [50]. However, no correlation between the SNP and PFS was eventually found [34]. In contrast, data on the circulating VEGF-A as a biomarker are more consistent, associating high baseline levels of plasma VEGF-A to a stronger effect of bevacizumab treatment. These data are supported by findings in the AVEREL [37] and the AVADO trial [31,51,52]. The data of AVEREL revealed that a high plasma concentration of VEGF-A at baseline was associated with a poorer prognosis, independent of the type of treatment. Moreover, the AVEREL study indicated that treatment with bevacizumab is more efficient in patients with high baseline VEGF-A concentrations than in patients with low VEGF-A concentrations, HR 0.70 *vs.* 0.83. *p* = 0.80 [37]. The same observations were found in the data of AVADO, in which the study populations were divided into four quartiles according to VEGF-A concentrations. The outcome showed non-significant improved hazard ratios with increasing VEGF-A-baseline levels [52]. Importantly, none of the described results were statistically significant, and future trials are required to investigate the role of VEGF-A plasma levels as a clinical predictor of outcomes in bevacizumab treatment [37]. To address these issues, a prospective trial focusing on the role of VEGF-A as a biomarker has recently started recruitment (clinicaltrials.gov ID: NCT01663727). The patients will be stratified according to their baseline levels of plasma VEGF-A before randomization to treatment of paclitaxel plus bevacizumab and paclitaxel plus placebo, respectively.

As briefly mentioned above, the development of hypertension during bevacizumab treatment has recently been demonstrated to be associated with an improved response. This statement emerges from a new retrospective study by Gampenrieder *et al.* [45] that shows that breast cancer patients developing hypertension during treatment had a more favorable outcome of PFS and two-year OS. Both results were significant: PFS 13.7 *vs.* 6.6 month, HR = 0.34, 95% CI = 0.23–0.49, *p* ˂ 0.001, and two-year OS 78% *vs.* 30%, HR = 0.20, 95% CI = 0.12–0.35, *p* ˂ 0.001 [45]. The population used in this analysis was heterogeneous, and hypertension is indeed not a desired effect of bevacizumab treatment. Nonetheless, this side effect of bevacizumab was associated with more favorable outcomes of anti-angiogenic therapy. A study by Keyhani *et al*. [53] focused on estimating the rate of angiogenesis in different breast cancer patient groups. The rationale for the analysis is that by defining which breast cancer patient groups have the biggest rate of angiogenesis, one can define which patients will benefit the most from angiogenic therapy. The study reported a correlation between tumor stage and simultaneous mutation of *HER2* and *TP53*, as well as a correlation between micro-vessel density and patient’s age and concluded that patients younger than 50 years had a higher rate of angiogenesis. Finally, neuropilin-1 and VEGFR-3 expression have also been evaluated as potential biomarkers. According to a few data of AVF2119g, low expression on neuropilin-1 seems to be associated with increased clinical benefits of bevacizumab [54]. Similar findings exist in trials of bevacizumab in gastric cancer and colorectal cancer [55,56]. In contrast, the AVADO trial showed inconsistent results regarding levels of neuropilin-1. High baseline levels of VEGFR-2 have demonstrated a more favorable outcome of bevacizumab than low baseline levels of VEGFR-2, but the difference in outcome was not significant [54]. In the case of more indications of a predictive role of VEGFR-2 expression, more trials focusing on VEGFR-2 as a biomarker would be relevant. To sum up, many different factors have been evaluated as biomarkers: HER2 status, genetic polymorphisms, plasma levels of VEGF-A, hypertension and age of patients. More research in this area is highly relevant, because patient benefits and the cost-effectiveness of anti-angiogenic drugs depend on the ability to target and monitor patients who will show the strongest response to anti-angiogenic therapy.

## 5. Conclusions and Future Developments

Anti-angiogenic agents impact and reduce the growth of malignant breast tumors. Until now, the use of agents targeting VEGF-driven angiogenesis in combination with standard protocols or in neoadjuvant therapy have shown modest clinical efficacy. Several contributing factors may explain the low efficacies obtained so far, including drug resistance, alternative triggers of angiogenesis, vessel co-option and the problem of identifying who will benefit from anti-VEGF therapy in breast cancer. However, the impact for angiogenic therapies in improving clinical outcomes is not yet clearly evaluated, but results from the TANIA and IMELDA trials published in 2014 [33,35] showed that continued second-line treatment with bevacizumab plus chemotherapy significantly improved PFS compared to bevacizumab alone, suggesting an improved efficacy of sustained anti-angiogenic treatment.

Furthermore, the IMELDA trial data suggest that an early switch to maintenance therapy might be beneficial, challenging the current treatment paradigms for HER2-negative metastatic breast cancer [57].

Breast cancers are heterogeneous in nature, which may account for much of the clinical variability observed from anti-angiogenic therapies. As these therapies are targeted towards specific signaling pathways, clinical success is highly dependent on *a priori* knowledge of the tumor responsiveness. Development of reliable biomarkers to identify which patients are likely to match the target pathology and to respond to anti-angiogenic therapy is a key factor. Molecular-based standards for evaluating a therapeutic index on an individualized basis before treatment with anti-angiogenic drugs may increase the efficacy substantially. To achieve this aim, translational research using genomic and proteomic technologies may be applied to samples of past and ongoing clinical trials to provide molecular signatures and formal sub-classification of tumors [58] that may help select/deselect appropriate treatments. Moreover, such research may also identify new target molecules and biomarkers and provide useful knowledge for designing future clinical trials. A promising candidate for future trials may be ellagic acid, which is shown to reduce VEGF-related processes significantly. Ellagic acid inhibits both VEGFR-2 and intracellular pathways and may be able to inhibit the growth of breast cancers [59]. Future therapeutic strategies utilizing multiple markers, from several sources, may help to predict the benefits of anti-angiogenic therapy more robustly than single-biomarker approaches [60].

Another aspect is that VEGF plays also an important immunological role [61]. It has been shown *in vitro* that mesenchymal stem cell-secreted IL-6 and VEGF may act as paracrine factors to sustain breast cancer cell migration [62]. In addition, it is known that programmed death-ligand 1 (PD-L1), which is expressed by endothelial cells, plays a key role in suppressing the immune system. Anti-PD-L1 together with anti-VEGF therapies are now being tested in clinical trials [61]. The future will show whether this combined approach has beneficial effects on the PFS and OS in breast cancer patients.

## Abbreviation

EC: Endothelial cell
ECM: Extracellular matrix
EMEA: European Medicines Agency
FDA: Food and Drug Administration
HER2: Human epidermal growth receptor 2
HER2-positive: Tumor shows overexpression of HER2-receptors
HER2-negative: Tumor does not show overexpression of HER2-receptors
HIF: Hypoxia induced factor
MAPK: Mitogen activated protein kinase
MMP: Matrix metalloproteinases
NPLD: Non-PEGylated liposome-encapsulated doxorubicin
ORR: Overall response rate
OS: Overall survival
pCR: Pathological complete response
PDGFB: Platelet-derived growth factor-b
PFS: Progression-free survival
PHD: Prolyl hydroxylase domain
PKC: Protein kinase C
PLGF-1/-2: Placental growth factor-1/-2
Raf-1: Proto-oncogene, serine/threonine kinase
SNP: Single nucleotide polymorphism
TIES: Trials to Investigate the Efficacy of Sorafenib
TNBC: Triple negative breast cancer: the tumor is HER2-negative, ER-negative and PR-negative
VEGF: Vascular endothelial growth factor
VEGF-A: Vascular endothelial growth factor A
VEGFR-2: Vascular endothelial growth factor receptor subtype 2
